# The New Advance at the International Congress of School Hygiene

**Published:** 1907-08-24

**Authors:** Lauder Brunton, James Kerr, E. White Wallis

**Affiliations:** President; Hon. General Secretary; Hon. General Secretary


### Editor's Letter-Box.

[Our Correspondents are reminded that prolixity is a great bar to publication, and that brevity of style and conciseness of statement greatly facilitate early insertion.]

### THE NEW ADVANCE AT THE INTERNATIONAL CONGRESS OF SCHOOL HYGIENE.

Sir,—As a slightly incorrect statement was published, it would be as well to explain in some detail the important new move in the matter of school hygiene which was taken at the closing meeting of the recent International Congress.

The permanent International Committee, consisting of about sixty members, selected from almost every country, has hitherto only met during Congresses. Arising out of the question of whether it would not be a proper thing to establish a bureau, with a permanent staff, library, and museum, and so on, in some central but neutral spot, such as a Swiss or Dutch town, it was decided, as explained by Drs. Mathieu, Burgerstein, and Kerr, that it would probably lead to greater progress if such bureau was not localised, but if each country had its own centre for the diffusion of knowledge, and to act as a clearing house in the matter of school hygiene statistics, laws, and regulations. Finally, to supervise in scientific matters, and generally to do all that is possible at all times or places to forward the human interests which are bound up in the special lines of knowledge included in school hygiene, the International Committee has formed a small Council.

This Council has all the powers of an ordinary committee. It can form sub-committees of experts on special inquiries. The usual committee procedure is to sit round a table and discuss matters, but this Council will deal with the various subjects that arise, submitting the different topics by correspondence, collating the answers, and finally making pronouncements in urgent matters after a meeting of the Council.

It is obvious that for efficiency such Council should be small, and yet have in it elements to secure permanence, and at the same time possibilities of slow but constant change. This has been done by deciding that it shall consist of the president of the past Congress, the president of the Congress which has just been held, and the president of the next Congress. Nine other members are to be elected, of whom three are to be from the country where the Congress was last held, and three from the country where it will be held next, three being selected from other lands.

Certain matters, for instance, will almost at once come under the consideration of this Council. Such might be quoted as :—

"The question of how medical inspection of schools can best be carried out with the maximum of efficiency and minimum of cost."

"The question of how far the laws of health can best be imparted to the coming generation, so that later they will know how to care for themselves and those dependent on them."

"The best systems or methods of physical training for both sexes at various ages."

"The feeding of children requiring proper nutrition, so that it shall be done without developing pauperism and with regard to those upon whom the cost falls."

These four matters are being dealt with practically in a great variety of ways, and this Council should be able to collect and analyse known facts to show which methods are best for any town or State.

It is obvious that information thus digested will have a very great value politically as well as educationally, and this Council may in time come to be officially regarded as quite analogous in matters of school hygiene to that other Congress of Peace now in session at The Hague.

Yours faithfully,
Lauder Brunton,
President.
James Kerr,
E. White Wallis,
Hon. General Secretaries.

THE HOSPITAL *August* 24, 1907.

